# Self-assessment in machines boosts human Trust

**DOI:** 10.3389/frobt.2025.1557075

**Published:** 2025-05-26

**Authors:** Dana Warmsley, Krishna Choudhary, Jocelyn Rego, Emma Viani, Praveen K. Pilly

**Affiliations:** Intelligent Systems Center, HRL Laboratories, Malibu, CA, United States

**Keywords:** machine self-assessment, trust calibration, trust in AI, autonomous systems, human-machine teaming

## Abstract

Low trust in autonomous systems remains a significant barrier to adoption and performance. To effectively increase trust in these systems, machines must perform actions to calibrate human trust based on an accurate assessment of both their capability and human trust in real time. Existing efforts demonstrate the value of trust calibration in improving team performance but overlook the importance of machine self-assessment capabilities in the trust calibration process. In our work, we develop a closed-loop trust calibration system for a human-machine collaboration task to classify images and demonstrate about 40% improvement in human trust and 5% improvement in team performance with trained machine self-assessment compared to the baseline, despite the same machine performance level between them. Our trust calibration system applies to any semi-autonomous application requiring human-machine collaboration.

## 1 Introduction

A lack of trust in autonomous systems continues to hinder their adoption and effectiveness, particularly in safety-critical, high-stakes applications. A clear signal of this problem is the high frequency of human takeover events when the system’s behavior does not match human expectations, or the human is insufficiently confident in its situational understanding. In this work, we develop a closed-loop trust calibration system that improves trust over time via reliable machine self-assessment. The system first assesses its own capability, or ability to complete a given task successfully, in real time. It then estimates the human’s trust and determines if there is a miscalibration between human trust and machine capability. If so, it takes some action to proactively align human trust with machine capability in real time, for example, by requesting human intervention or showing a confidence score as a signal of its capability for the human. In doing so, the system can reduce instances of over-reliance that occur when the human trusts the machine to perform a task it is not capable of, and instances of under-reliance when the human does not trust the machine to perform tasks that it is capable of.

The contributions of this work are three-fold: (1) Developed a closed-loop trust calibration system that leverages real-time trust prediction, machine self-assessment, and a dynamic reasoning model that determines the best machine action to encourage trust calibration. (2) Empirically compared the cumulative trust of human participants in machines with learned self-assessment to that in machines without it. (3) Developed a paradigm to rigorously assess the effects of trust modeling and self-assessment in machines on human trust for operationally relevant contexts.

## 2 Prior work

Early work in trust calibration focused on transparency, which involved consistently offering uncertainty information, confidence estimates, or system reliability to encourage appropriate trust in the machine (Mercado et al., 2016; Yang et al., 2017). More recently, efforts shifted to *adaptive* trust calibration, where the system either selectively determines when to provide these information cues to calibrate trust or adapts its behavior to the human. Adaptive trust calibration efforts are of wide interest since having the human continually monitor information cues can increase workload (Kunze et al., 2019; Akash et al., 2020), and adaptation allows for personalization to the individual. See Wischnewski et al. (2023) for a pertinent survey on trust calibration. Here, we highlight some recently developed adaptive trust calibration systems relevant to our study.

Okamura and Yamada (2020) developed a framework for offering trust calibration cues when over- and under-trust were detected. Over-trust occurs when the human incorrectly believes the machine will perform the task better, and under-trust happens when the human incorrectly believes the machine will perform worse. They found that adaptively offering the cues improved trust and team performance. Fukuchi and Yamada (2023) presented the Predictive Reliance Calibrator (Pred-RC) method to adaptively decide when to provide reliance calibration cues (confidence information), where reliance is considered an observable trust-related behavior. If Pred-RC determined that the probability of reliance was higher with the cue than without and the probability of machine success was high, the cue was shown to encourage reliance. Pred-RC reduced the number of cues needed while avoiding over/under-reliance on the machine.

Chen et al. (2018) learned a Partially Observable Markov Decision Process (POMDP) model that used inferred trust levels to determine what robot actions would maximize team performance. In a table-clearing task, the robot learned to build human trust by clearing low-risk objects (high-risk objects) when trust was low (high). Further, Akash et al. (2020) developed a POMDP model of the effects of automation reliability, transparency, scene complexity, gaze behaviors, and reliance on human trust and workload dynamics in Level 2 driving scenarios. The model was leveraged to use current human trust and workload levels to calculate the optimal level of system transparency necessary to calibrate trust in real time.

We model our experiments after the study of Ingram et al. (2021), which investigated compliance, transparency, and trust calibration in an autonomous image classifier. In particular, they tested whether showing the classifier’s confidence values would increase trust in it. They found that trust was largely based on system performance (accuracy) and did not increase as a result of presenting system confidence information to the human. We hypothesize that they did not see an overall increase in trust because they used the predicted class probability as a proxy for system confidence, which has shown to be a poor method for self-assessment (Guo et al., 2017). Accurate machine self-assessment is critical since cues intended for trust calibration can worsen it if they are not reliable (Yeh and Wickens, 2001).

Going beyond prior work, we developed a closed-loop trust calibration system that adaptively asks for human assistance during the image classification task based on not only self-assessed machine capability but also predicted human trust level. We placed special emphasis on accurate machine self-assessment in encouraging appropriate trust in and reliance on automation and showed in experiments that improved self-assessment boosts overall trust in the machine, reduces over- and under-reliance behaviors, and increases team performance.

## 3 Closed-loop trust calibration system

In what follows, we describe the three major components of our closed-loop trust calibration system that was developed for human-machine teaming in the image classification domain. The first component is a machine self-assessment module that estimates the image classifier’s confidence in its label, independent of class probabilities. The second is a real-time human trust prediction model. The third is a dynamic reasoning component that, given the classifier’s confidence and the human’s trust level, determines whether or not to ask the human for assistance. These components were trained and evaluated using data from two rounds of experiments in which humans worked with semi-autonomous image classifiers to classify 50 images and rated their trust after assessing machine performance for each image (details in Section 4). We present component-specific results in the following sections and team performance-related results later in the article.

### 3.1 Machine self-assessment

Neural networks trained as image classifiers typically have a final layer of neurons, where each neuron corresponds to a class in the dataset. The neuron with the highest probability (after softmax operation) is chosen as the image label. A widely used baseline for confidence in that label is its corresponding probability. In practice, this probability is not constrained to correlate with the accuracy of the predicted label, leading to overconfident errors and underconfident predictions. Indeed, softmax probabilities are known to be non-calibrated, sensitive to adversarial attacks, and inadequate for detecting out-of-distribution examples (Guo et al., 2017; Corbiere et al., 2019).

**FIGURE 1:**
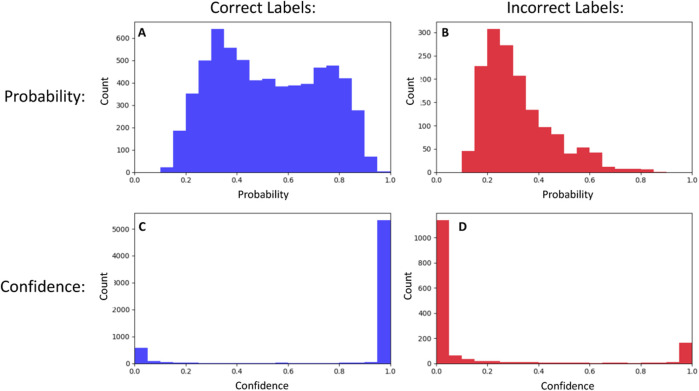
A neural network image classifier can use the probability that an image belongs to a label/class (top row) as a measure of confidence. We show the distribution of these probabilities for correct **(A)** and incorrect **(B)** labels for a subset of the images in the STL-10 dataset. These distributions have significant overlap, so probability is a poor indicator of correctness. In comparison, the self-assessment method for learning confidence scores that correlate with accuracy produces distributions that are much more distinct, with values close to one for correct labels **(C)** and close to 0 for incorrect labels **(D)**.

Corbiere et al. (2019) introduced a new confidence metric based on the True Class Probability (TCP), which is the probability of the correct class regardless of whether that class was chosen as the predicted label by the classifier. As it is not known at test time, they implemented a separate neural network (called ConfidNet) that operates on high-level features extracted by the classifier neural network and learns to estimate the TCP during training. Webb et al. (2021) provided an alternate method to train the confidence neural network to output “correctness” instead of TCP. That is, the neural network is trained to output a value of one if the label is correct and 0 otherwise. We follow this method for machine self-assessment in our work. In Figure 1, we show a comparison of learned self-assessment to the baseline use of probability on a subset of images from the STL-10 dataset (Coates et al., 2011). As expected, it outputs predominantly low values for incorrect labels and predominantly high values for correct labels. Moving forward, we use the terms “Unaware Classifier” for the image classifier that uses class probability as the confidence estimate and “Aware Classifier” for the image classifier that uses learned self-assessment (Webb et al., 2021) since the network trained on top of the image classifier is aware of the latter’s capability to classify images. The confidence metric itself is task-agnostic but requires a training scheme that is adapted to the specifics of the learning task. For instance, self-assessment for a reinforcement learning-based agent is a dynamic confidence metric that predicts the probability of success in a given episode over time steps (Valiente and Pilly, 2024).

Note that ConfidNet is not the only method for achieving accurate self-assessment. Other effective methods, such as Monte Carlo dropout, ensemble modeling, and temperature scaling, can be employed depending on the specific application. Temperature scaling learns a global temperature parameter to adjust the predicted probability distributions for overall more reliable confidence scores (Pereyra, 2017); however, it is not designed to provide input-specific confidence. Monte Carlo dropout (Gal, 2016) and ensemble modeling (Lakshminarayanan, 2017) offer more principled approaches to uncertainty estimation that can adapt to different inputs, but they typically incur higher computational costs. Monte Carlo dropout involves performing multiple stochastic forward passes through the network, with dropout applied independently in each pass, to produce a distribution over predictions. Ensemble modeling, on the other hand, requires training and running several independent models. Ultimately, we selected ConfidNet for its low computational cost during both training and inference, as well as its potential for robust generalization to out-of-distribution data.

### 3.2 Trust prediction model

In real-world applications, humans will not regularly provide feedback for the machine to assess the need for trust calibration. Human trust must be predicted from potentially sparse information. Earlier approaches utilized rule-based and statistical models, while recent research has shifted towards Long Short-Term Memory (LSTM) networks. These models are better suited for capturing temporal dependencies, thereby improving predictive accuracy (Olabiyi et al., 2017). Our system employed an LSTM network and was trained using data collected from the first round of experiments to predict human trust based on readily available inputs—the ground truth accuracy of the image classifier (since humans reviewed machine performance in each trial and could intervene if needed), the classifier’s confidence in its label, and the compliance of the participant (whether a participant chose to assist (not assist) the classifier when assistance is (is not) requested). The model was evaluated on a validation set, for which it obtained an overall Mean Squared Error (MSE) of 1.67 on a 0–100 scale.

**FIGURE 2:**
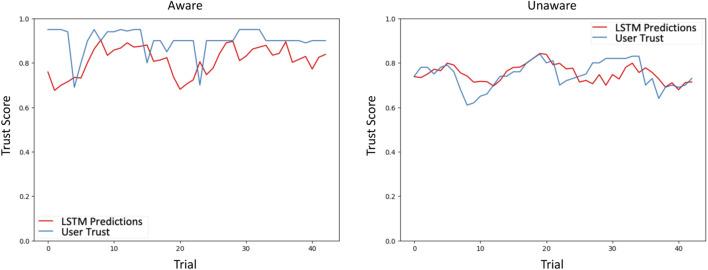
The LSTM Trust Prediction Model predicts human trust at each time step given information about the machine (accuracy, confidence) and human behavior (reliance). We show illustrative results for a single participant from the second round of experiments. There are only 43 trials in the plots because the model used a sliding window of seven time steps during training.

The model was then employed to predict human trust in the second round of experiments, for which it obtained an MSE of 3.39 for predicting trust in the Unaware classifier and 3.21 for predicting trust in the Aware classifier, resulting in an overall MSE of 3.3. Figure 2 shows the prediction results for a single participant. Note that for our purposes, we primarily needed the model to predict general trust trends, not precise trust levels.

### 3.3 Dynamic reasoning model

The Dynamic Reasoning Model determines when to ask for assistance based on machine confidence and predicted human trust. In the first round of experiments, the model used a dynamic threshold. Machine confidence values below the threshold resulted in the machine asking for assistance. After initializing this threshold at 50% for the first trial of the experiment, the threshold was adjusted according to the compliance of human actions with the machine’s request for assistance. If the machine asked for assistance but assistance was not given, the threshold was decreased by five points, thus triggering requests for assistance at relatively lower confidence values in future trials. If the machine did not ask for help but its human partner intervened anyway, the threshold was increased by five points, resulting in requests for assistance at relatively higher confidence values in future trials. By dynamically changing the threshold for the machine to request assistance in this manner, we calibrate human trust based on machine capability in real time.

In the second round of experiments, we replaced the threshold rule with a model trained on data from the first round. We hypothesized that a model with knowledge of trust levels, machine confidences, and instances of human intervention would be able to determine when assistance is both needed by the machine and is likely to be given by its human partner. We trained a three-layer feedforward neural network to predict whether a human would assist the machine given the human’s predicted trust level from the previous trial and the machine’s confidence in the current trial. When tested on a held-out set of examples from the first round of experiments, our model reached an accuracy of 83.94% for the Aware classifier and 82.91% for the Unaware classifier.

## 4 Materials and methods

During experiments, human participants were asked to team with an autonomous image classifier to complete an image classification task to maximize team performance while minimizing their effort under time constraints. Each participant engaged in two sessions - one with the Unaware classifier (using softmax probability for the predicted label) and the other with the Aware classifier (using learned self-assessment). We hypothesized that improved self-assessment capabilities would lead to improved overall trust and team performance since humans are more likely to trust and appropriately rely on a machine that knows when it can and cannot complete a task. We offer the following hypotheses:

Hypothesis 1 We will observe increased overall trust in the Aware classifier, despite equal machine performance (classification accuracy).

Hypothesis 2 Teaming with the Aware classifier will result in a larger reduction in human over- and under-reliance on the machine since improved self-assessment means the machine is better able to ask for assistance when needed.

Hypothesis 3 Teaming with the Aware classifier will result in better team performance (classification accuracy). Reduction in over- and under-reliance behaviors reduces both machine and human error.

### 4.1 Experimental paradigm

**FIGURE 3:**
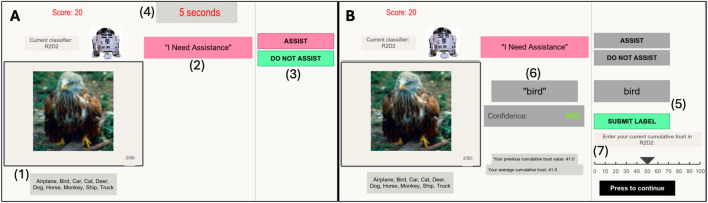
The GUI at different points during an experimental trial: **(A)** shows the GUI at the beginning of the trial. The participant must use the information of (1) the image and potential classes and (2) the machine’s request for assistance (or lack thereof) to first determine whether they will (3) assist the machine or not within a time frame of 5 seconds (4). If they decide to assist the machine **(B)**, they are first asked to enter a label and submit it (5). Note if the human does not decide to assist, the GUI does not ask for the label. The machine’s label and confidence score then appear for the human’s review (6), and the human must use this information to provide a score for their cumulative trust in the machine (7).

Image Classification Task: During the image classification task, a Graphical User Interface (GUI) built using the PsychoPy Python library served as the point of interaction between the participants and image classifiers. In a single session of the main task, the participants were presented with 50 images consecutively. At the start of each trial (Figure 3A), the participants were shown the image, the name of the classifier (R2D2 or Wall-E), the current team performance score, the classifier’s request or refusal for assistance (“I Need Assistance” or “I Do Not Need Assistance”), a countdown clock for the time remaining to decide whether to assist, and “Assist” and “Do Not Assist” buttons. They were given 5 seconds to decide about assistance, after which the machine submitted its label as the team label. If the participants chose to assist, they were prompted to enter a label into a text box to stand as the team label. Participants did not have to comply with machine requests for help and also had the option to assist even when the machine did not ask for help.

After submitting the team label (Figure 3B), the GUI displayed an updated team performance score, the classifier’s label for the image, and the classifier’s confidence in that label (with the color of the confidence estimate shown ranging from red to green on a 0–100 scale). The team performance score was a running average across trials that started at 100 points at the beginning of the experiment and was penalized in subsequent trials for over and under-reliance behaviors. In each trial, the team would receive a score of 0 points if either the human decided to take control from the machine and label the image even though the machine would have labeled the image correctly, or the human decided not to take over and allowed the machine to submit an incorrect label. Otherwise, they received 100 points for the trial. Finally, having reviewed the classifier’s label and confidence, the participants were asked to report their cumulative trust in the classifier based on their overall experience with it since the start of the session. Previous and average cumulative trust levels were displayed to aid the participants in keeping track of their trust development and to encourage them to view their current trust rating as cumulative.

Procedure: Participants were recruited from HRL Laboratories using flyers and received monetary compensation upon completion of the study. All subjects provided signed informed consent to participate in the study, which was reviewed and approved by the Institutional Review Board of WCG Clinical Services. Participants then completed an adaptation session, which involved filling out a pre-experiment survey and reading through instructional slides describing the task and GUI. Instructions informed participants that they would team with a machine partner to classify images and that the machine would ask for assistance when it thinks that its label could be wrong. Participants were instructed to report overall trust in the machine at the end of each trial, after viewing the machine’s label and confidence estimate for that trial’s image. Participants then completed a demo to ensure they understood the task and how to interact with the GUI. They then performed the core image classification task over two main sessions for the two classifiers, with a post-experiment survey to assess their overall trust in the respective classifier and gain insight into their impression of the classifier’s performance.

Pre- and Post-experiment Surveys: The pre-experiment survey collected demographic information, including age, race, gender, country of birth, education, and prior experience with image classifiers and semi-autonomous systems. Participants also completed the mini-IPIP scale to assess the Big Five personality traits and a validated propensity-to-trust-automation survey (Yang et al., 2023) before engaging with the image classifier. For both, participants rated each statement in the survey on a five-point Likert scale (strongly disagree to strongly agree). The post-experiment survey (Figure 5) was presented to participants after each of the Aware and Unaware sessions to gauge their overall experience with each classifier. We used a validated trust-in-automation survey (Yang et al., 2023), replacing the term “decision aid” with “classifier” for specificity. Participants rated each entry on a 1–10 scale.

Images: We used the STL-10 dataset, which consists of 10 classes of objects (airplane, bird, car, cat, deer, dog, horse, monkey, ship, truck). To create uniformity in our experiments, we created two groups of 50 hand-picked images that were high contrast, had clear, singular objects, and were not used to train the self-assessment model. We made the selection such that the class distribution and the accuracy within each class were preserved. For example, the classifier we used in the experiments accurately classified airplanes only 50% of the time, while it accurately classified ships 99% of the time. We ensured this asymmetry was reflected in the image groups that the participants viewed.

Experimental Design: We performed two rounds of experiments with eight participants each. In both rounds, we used a 2 × 2 × two counterbalanced within-subjects design where participants were exposed to the Aware and Unaware classifiers (both with 80% accuracy), the name of the classifier (Wall-E or R2D2), and the set of 50 images (Group 1 or Group 2). The two rounds differed in terms of the Dynamic Reasoning model deciding when to ask for assistance (Section 3.3).

## 5 Results

### 5.1 Experiment results - first round

Paired t-tests were used to determine if there were significant differences (p<0.05) in reported trust, over- and under-reliance on the machine, and team performance between the Aware and Unaware classifiers.

**FIGURE 4:**
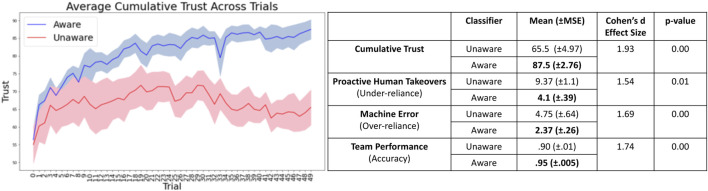
In the first round of experiments, we showed that the closed-loop trust calibration system with improved self-assessment (the Aware classifier) resulted in increases in human trust and team performance, and a decrease in over- and under-reliance. In particular, improved self-assessment led to improved trust in the machine by about 34%.

Cumulative Trust: Participants reported about 34% higher trust in the Aware classifier as compared to Unaware (Figure 4, left). This result was statistically significant (p=0.0002) with a large effect size (Cohen’s d = 1.93) and supports *H1* (Figure 4, right).

Over- and Under-reliance: Under-reliance occurs when the participant assists the machine even though it is capable of correctly labeling the image (proactive human takeover). Over-reliance occurs when the participant does not assist even though the machine cannot correctly label the image. Sessions with the Aware classifier resulted in fewer proactive takeovers (4.1 vs 9.37 takeovers) and fewer instances of over-reliance (2.37 vs 4.75 machine errors without human assistance), on average, as compared to Unaware. These results were statistically significant with a large effect size as well and support *H2* (Figure 4, right).

Team Performance: Team performance when participants worked with the Aware classifier (95% classification accuracy) surpassed that when working with the Unaware classifier (90% classification accuracy). This result was statistically significant with a large effect size as well and supports *H3* (Figure 4, right).

**FIGURE 5:**
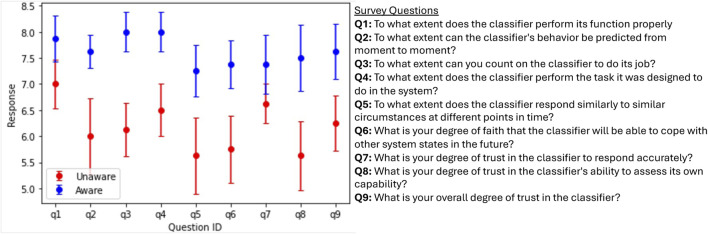
At the end of the first round of experiments, we gave a post-experiment survey to better understand participants’ experiences with and trust in the image classifiers. The graph (left) shows the mean participant response to the nine post-experiment survey questions (right) for both the Aware and Unaware classifiers. Results show that participants had an overall higher trust in and preference for the Aware classifier.

Figure 5 shows the mean participant response to questions in the post-experiment survey. The purpose of surveying participants after each session was to (1) gauge their overall trust in the machine once the task was completed, (2) validate the self-reported trust values observed during the experiment, and (3) understand how different aspects of the classifiers affected their trust. Overall, participants perceived the Aware classifier as higher performing than the Unaware classifier. Question 9, in particular, validates the results in Figure 4, indicating that participants did indeed have higher trust in the Aware classifier. Question eight supports our hypothesis that the difference in self-assessment capabilities largely drove this difference in perceived trust.

### 5.2 Experiment results - second round

**FIGURE 6:**
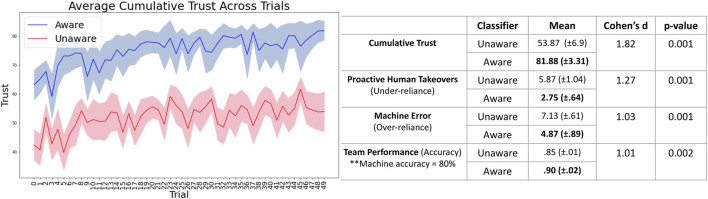
In the second round of experiments as well, we obtained statistically significant results of increased human trust (by about 52%), reduced over- and under-reliance, and improved team performance.

The second round involved using real-time prediction of trust instead of relying on compliance of human intervention and a neural network to determine when to ask for assistance instead of a threshold-based rule. Results of this round of experiments indicate that the first round’s results hold even when the system does not rely directly on reported trust in each trial (Figure 6). This suggests that our system could apply to more realistic situations in which the machine operates with sparse human feedback.

## 6 Discussion

As previously mentioned, we most directly compare our work to Ingram et al. (2021), which found that presenting the probability of the assigned label as a confidence score did not increase trust in the image classifier. In contrast, by employing learned self-assessment confidence scores that better correlate with machine accuracy, we observed an increase in overall trust in the machine by about 34% and 52% by the end of the first and second rounds, respectively. This highlights that confidence scores and/or other competence metrics must be accurate to be effective in fostering trust. It is important to distinguish machine self-assessment from machine explainability and transparency, which aim to make the machine’s internal processes more interpretable and help humans understand the reasoning behind a decision regardless of its correctness. While both concepts are valuable, we have focused only on machine self-assessment in this study.

While we observe an increase in human trust in the machine, our goal is not merely to inflate trust, as this could lead to over-reliance of the human on the machine in future tasks. The observed increase in overall trust is, in fact, a byproduct of the trust calibration process. Our experiments demonstrate that adaptively providing cues to humans for real-time trust calibration (through intervention requests) is effective in fostering appropriate reliance *during* the task while improving overall team performance and trust by the end of the task. A machine capable of assessing and communicating its ability in real time is more likely to be trusted and more likely to be adopted for long-term use. Although our study is consistent with prior research (Section 2), our main contribution lies in the closed-loop trust calibration system that takes both self-assessed machine capability and predicted human trust level into account.

Our study has several limitations. Firstly, our results are based on a relatively small sample of 16 engineers and scientists from HRL Laboratories, all of whom had prior experience with artificial intelligence (AI) and autonomous systems. Given that established trust models highlight experience with automation as a significant factor influencing trust (Mayer et al., 1995; Hoff and Bashir, 2013)), our results may not generalize to a population that is less knowledgeable about or experienced with AI. Furthermore, these models highlight other factors that influence trust, including human factors (e.g., culture, personality, workload), machine factors (e.g., ability, benevolence, integrity), and situation-specific factors (e.g., task difficulty, risk). While we do not consider these factors in this study, a compelling future experiment would investigate their role in trust dynamics and their interaction with self-assessment methods in human-machine teams.

Another limitation is that our experimental scenario was relatively less complex compared to real-world applications that would most benefit from autonomous systems, such as autonomous vehicles and robots. In particular, the image classification task was not dynamic, and both the human and machine were essentially performing the same task. Typically, autonomous machines offer the greatest advantage when they allow the human to multitask and intervene only when necessary. Also, our experimental design allowed for continuous human review of the machine’s capability, which is often not feasible in dynamic, real-world settings. While we hypothesize that our results would extend to more complex collaborative tasks, future work is needed to confirm this.

Finally, we acknowledge that there are currently no known methods that can provide guarantees for the accuracy of machine self-assessment, especially for complex autonomous systems encountering novel situations. We were also able to test only two contrasting self-assessment methods, one learning-based and the other not. This highlights the need for responsible human oversight of AI for high-risk applications, albeit with potentially reduced cognitive workload, as well as the importance of integrating self-assessment with offline human-machine co-training (Miller et al., 2023). The latter will facilitate the continuous calibration of human expectations regarding the machine’s capability and self-assessment abilities, both before and after each collaborative task. We reserve the exploration of these aspects for future work.

## 7 Conclusion

In this work, we developed a closed-loop trust calibration system for human-machine collaboration in the image classification task that included a real-time human trust prediction model, a machine self-assessment model, and a dynamic reasoning model that determined when the machine should ask for human assistance to calibrate trust. We performed human experiments to highlight the importance of accurate self-assessment for trust calibration. Specifically, we showed that improved self-assessment capabilities result in increased overall trust in the autonomous image classifier, reduced over- and under-reliance behaviors on the part of the human, and improved overall team performance in the classification task. In future work, we would like to extend our experiments such that (1) we require multi-tasking on the part of the human, (2) we use more dynamic scenarios where the human may also be uncertain about their ability to accomplish the task, and (3) we incorporate pre-task training for the human to learn more about the machine before engaging in the task. We expect these extensions will increase the applicability of our trust calibration system to more complex, real-world scenarios.

## Data Availability

The datasets presented in this article can be made available upon approval of the HRL Laboratories Legal Department. Requests to access the datasets should be directed to dmwarmsley@hrl.com.
