# Supplementary material for: Self-assessment in machines boosts human Trust
Source: Front Robot AI. 2025 May 26;12:1557075. doi: 10.3389/frobt.2025.1557075 (PMC12146354; doi:10.3389/frobt.2025.1557075)
Supplement: Supplementary file 1 [file Supplementaryfile1.pdf]

Supplementary Material

1 TRUST CALIBRATION SYSTEM MODELING

1.1 Machine Self-Assessment

1.1.1 Image Classifier

For image classification, we start with a pre-trained EfficientNet-B0 model (Tan and Le, 2019). To use it as an encoder, we remove its final three layers, leaving a 1280-neuron output layer that serves as our latent space. To build the classifier, we add a single-layer neural network with linear units that maps the latent space to N output neurons, where N represents the number of classes. This classifier uses the softmax function, assigning the class with the highest softmax probability as the image label. The classifier is trained using batches of 16 images from the training set until it achieves 80% accuracy on the test set. Training employs the cross-entropy loss function and the Stochastic Gradient Descent (SGD) optimizer with a learning rate of 0.01, momentum of 0.9, and weight decay of 0.0001.

1.1.2 Machine Self-Assessment

For self-assessment, we implement a secondary neural network based on ConfidNet (Webb et al., 2021) that uses the 1280-dimensional latent representation of each image as input. This network comprises four fully connected layers with 400 neurons each, followed by a single-neuron layer that outputs the confidence score. During training, the classifier's weights remain frozen, so back-propagation updates the weights only in ConfidNet. To ensure unbiased confidence estimates, each training batch includes an equal number of correctly and incorrectly labeled samples. We train the network using the binary cross-entropy loss function and the Adam optimizer, with the learning rate set to 0.01.The model was trained for 50 epochs, with a batch size equal to the one used for classifier training.

1.2 Trust Prediction Model

The model comprises two bidirectional LSTM layers (Olabiyi et al., 2017) with 64 neurons each, followed by a fully connected layer with eight neurons and a single-neuron layer that estimates human trust on a 0-100 scale. The hidden layers use the ReLU activation function, while the final layer employs a linear activation function. To improve generalization, 3% dropout is applied to the outputs of the first LSTM layer and the fully connected layer. A sliding window of seven time steps is used during training to capture temporal dependencies. Among the input features, the classifier's confidence scores were scaled from 0-100 to 0-1. The other features (namely, ground truth and compliance) were represented as binary values.

To prepare the data for training the model, we grouped each image with its preceding seven time steps. Each participant's data was split into subsets of seven images, preserving the temporal order within the subsets. The target trust level was extracted from the image immediately following each subset. This resulted in a training set with the shape $16 \times 43 \times 7 \times 3$, where the first dimension represents participants (8 participants with 2 sessions each), the second dimension is the number of subsets per participant (50 images per session minus the 7 time steps), the third dimension is the sliding window size (7 time steps), and the fourth dimension represents the number of input features for each image. For training, the first two dimensions were flattened, resulting in a dataset of shape $688 \times 7 \times 3$.

Model weights were initialized using an orthogonal matrix obtained from the decomposition of a matrix of random numbers sampled from a normal distribution. We trained the model using a batch size of 43, allowing the LSTM layers to reset their internal states between participants and classifiers. This approach enabled the model to learn temporal relationships specific to each participant and classifier, rather than learning the average predictive model across all experimental sessions. Training employed the MSE loss function, minimized using the Adaptive Gradient Descent (AdaGrad) optimizer. The model was trained for 300 epochs with early stopping and learning rate decay as regularization techniques.

1.3 Dynamic Reasoning Model

The model was trained to minimize binary cross-entropy loss function with a learning rate of 0.001, across 5000 epochs with batches of size 32 and data sampled evenly between the Aware and Unaware classifiers from the first round of experiments.

REFERENCES

Olabiyi, O., Martinson, E., Chintalapudi, V., and Guo, R. (2017). Driver action prediction using deep (bidirectional) recurrent neural network. *arXiv preprint arXiv:1706.02257*

Tan, M. and Le, Q. V. (2019). EfficientNet: Rethinking model scaling for convolutional neural networks. *arXiv preprint arXiv:1905.11946*

Webb, T. W., Miyoshi, K., So, T. Y., Rajananda, S., and Lau, H. (2021). Performance-optimized neural networks as an explanatory framework for decision confidence. *bioRxiv preprint bioRxiv:2021.09.28.462081*
